# Crystal structure of bis­(propane-1,3-di­ammonium) hexa­fluorido­aluminate fluoride trihydrate

**DOI:** 10.1107/S1600536814018844

**Published:** 2014-08-30

**Authors:** I. Abdi, K. A. Al-Sadhan, A. Ben Ali

**Affiliations:** a Université de Carthage, Faculté des Sciences de Bizerte, UR11ES30, 7021 Jarzouna, Tunisia; b Department of Chemistry, Girls College of Science, University of Dammam, PO Box 838, Dammam 31113, Saudi Arabia

**Keywords:** crystal structure, hexa­fluorido­aluminate, hybrid aluminates, hydrogen bonding

## Abstract

The title compound, (C_3_H_10_N_2_)_2_[AlF_6_]F·3H_2_O, was obtained using the solvothermal method with aluminium hydroxide, HF and propane-1,3-di­amine as precursors in ethanol as solvent. The structure consists of isolated [AlF_6_]^3−^ octa­hedra, diprotonated propane-1,3-di­amine cations [(H_2_dap)^2+^], free fluoride ions and water mol­ecules of solvation. The Al—F bond lengths in the octa­hedral [AlF_6_]^3−^ anions range from 1.7690 (19) to 1.8130 (19) Å, with an average value of 1.794 Å. Each [AlF_6_]^3−^ anion is surrounded by three water mol­ecules and by six diprotonated amine cations. The ‘free’ fluoride ion is hydrogen bonded to four H atoms belonging to four dications and has a distorted tetra­hedral geometry. The three water mol­ecules are connected by hydrogen bonds, forming trimers that connect the AlF_6_ octa­hedra and dications into a three-dimensional framework.

## Related literature   

For general background to hybrid aluminates, their syntheses and applications, see: Ben Ali *et al.* (2007 ▶, 2009 ▶); Lhoste *et al.* (2009 ▶); Adil *et al.* (2010 ▶); Martineau *et al.* (2012 ▶); Cadiau *et al.* (2013 ▶). For a review of hydrogen-bonding inter­actions, see: Steiner (1998 ▶).
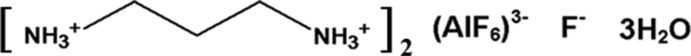


## Experimental   

### Crystal data  

(C_3_H_10_N_2_)_2_[AlF_6_]F·3H_2_O
*M* *_r_* = 366.31
Triclinic, 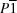

*a* = 9.825 (2) Å
*b* = 9.974 (3) Å
*c* = 10.697 (2) Å
α = 70.01 (2)°
β = 67.89 (2)°
γ = 59.77 (1)°
*V* = 823.8 (3) Å^3^
*Z* = 2
Mo *K*α radiation
μ = 0.21 mm^−1^
*T* = 298 K
0.61 × 0.13 × 0.08 mm

### Data collection  

Siemens AED2 diffractometer
Absorption correction: gaussian (*SADABS*; Sheldrick, 1996 ▶) *T*_min_ = 0.968, *T*_max_ = 0.985
3411 measured reflections
3411 independent reflections
3046 reflections with *I* > 2σ(*I*)
3 standard reflections every 120 min intensity decay: 4%

### Refinement  

*R*[*F* ^2^ > 2σ(*F* ^2^)] = 0.059
*wR*(*F* ^2^) = 0.183
*S* = 1.05
3411 reflections
215 parameters
H atoms treated by a mixture of independent and constrained refinement
Δρ_max_ = 0.62 e Å^−3^
Δρ_min_ = −0.54 e Å^−3^

### 

Data collection: *STADI4* (Stoe, 1998 ▶); cell refinement: *STADI4*; data reduction: *X-RED* (Stoe, 1998 ▶); program(s) used to solve structure: *SHELXS97* (Sheldrick, 2008 ▶); program(s) used to refine structure: *SHELXL97* (Sheldrick, 2008 ▶) and *WinGX* (Farrugia, 2012 ▶); molecular graphics: *DIAMOND* (Brandenburg, 2001 ▶) and *ORTEP-3 for Windows* (Farrugia, 2012 ▶); software used to prepare material for publication: *enCIFer* (Allen *et al.*, 2004 ▶).

## Supplementary Material

Crystal structure: contains datablock(s) I, global. DOI: 10.1107/S1600536814018844/cq2011sup1.cif

Structure factors: contains datablock(s) I. DOI: 10.1107/S1600536814018844/cq2011Isup2.hkl

**Fig. 1.**
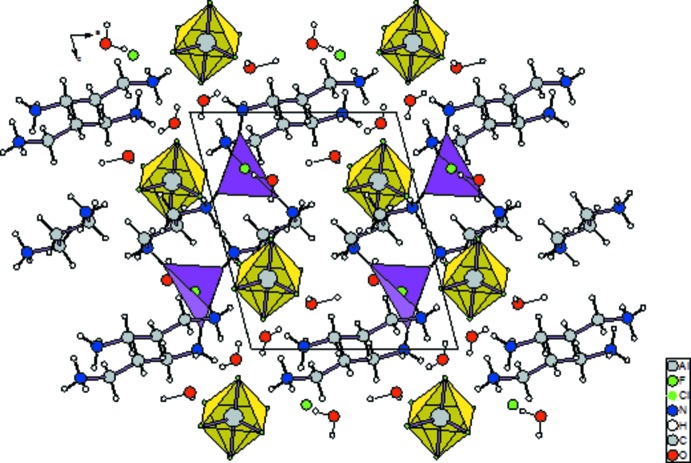
View of the structure of (I) along the [010] axis.

. DOI: 10.1107/S1600536814018844/cq2011fig1.tif
View of the structure of (I) along the [010] axis.

**Fig. 2.**
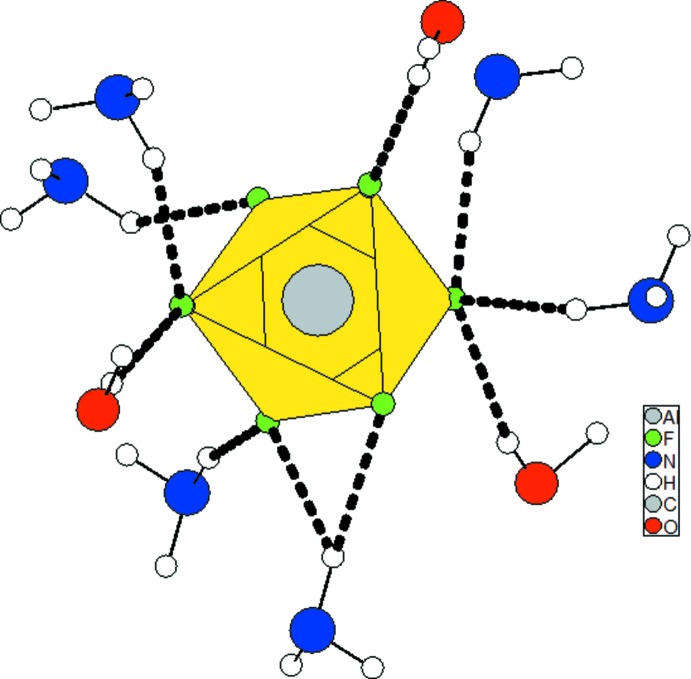
The environment of the AlF6 octahedron.

6 . DOI: 10.1107/S1600536814018844/cq2011fig2.tif
The environment of the AlF_6_ octa­hedron.

**Fig. 3.**
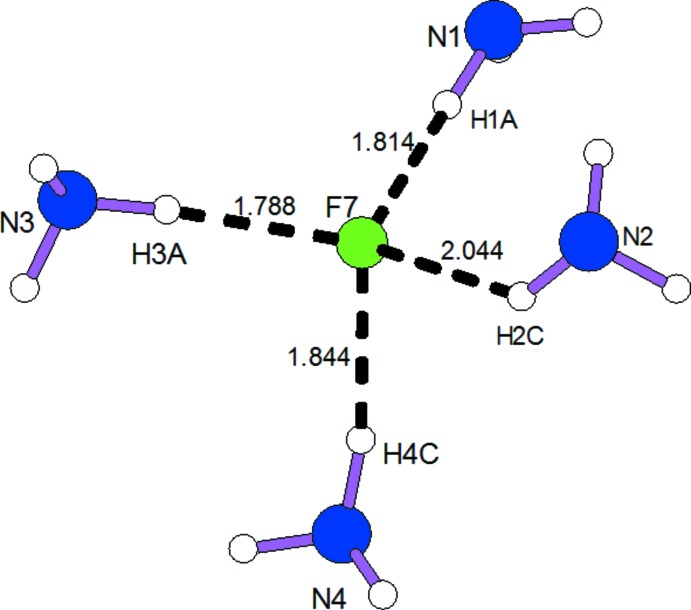
The environment of the isolated fluoride anion.

. DOI: 10.1107/S1600536814018844/cq2011fig3.tif
The environment of the isolated fluoride anion.

**Fig. 4.**
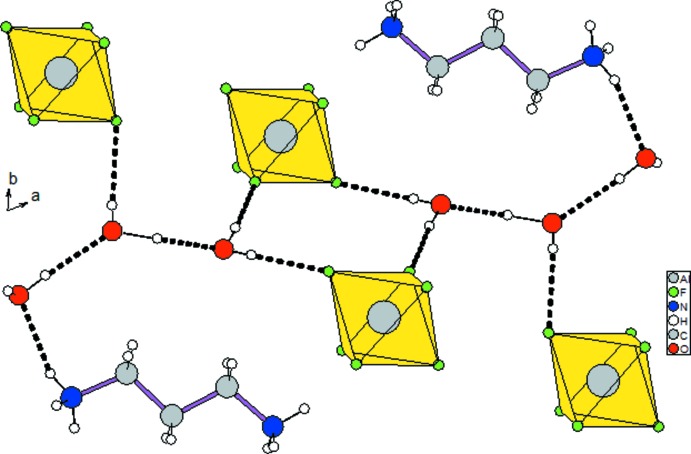
The environment of water molecules

. DOI: 10.1107/S1600536814018844/cq2011fig4.tif
The environment of water mol­ecules

CCDC reference: 1012356

Additional supporting information:  crystallographic information; 3D view; checkCIF report

## Figures and Tables

**Table 1:** Hydrogen-bond geometry (Å, °)

*D*—H⋯*A*	*D*—H	H⋯*A*	*D*⋯*A*	*D*—H⋯*A*
N1—H1*A*⋯F7	0.89	1.81	2.696 (3)	171
N1—H1*B*⋯F3^i^	0.89	1.79	2.663 (3)	166
N1—H1*C*⋯F2^ii^	0.89	2.08	2.826 (3)	141
N1—H1*C*⋯F3^ii^	0.89	2.09	2.796 (3)	136
N2—H2*A*⋯F4^iii^	0.89	2.08	2.657 (3)	122
N2—H2*B*⋯O*W*2^iv^	0.89	2.11	2.804 (3)	134
N2—H2*C*⋯F7^v^	0.89	2.04	2.724 (3)	132
N3—H3*A*⋯F7	0.89	1.79	2.677 (3)	176
N3—H3*B*⋯F5	0.89	2.00	2.792 (3)	148
N3—H3*C*⋯F5^iii^	0.89	1.89	2.753 (4)	162
N4—H4*A*⋯F2^ii^	0.89	1.90	2.757 (3)	161
N4—H4*B*⋯F1^vi^	0.89	1.92	2.776 (4)	162
N4—H4*C*⋯F7^ii^	0.89	1.84	2.724 (3)	169
O*W*1—H11⋯F5	0.92	1.85	2.743 (3)	161
O*W*1—H12⋯O*W*3^vii^	0.95	1.85	2.789 (5)	173
O*W*2—H21⋯F1	0.74	2.27 (5)	2.943 (3)	152 (5)
O*W*2—H22⋯O*W*1^viii^	0.82 (7)	1.98	2.785 (5)	170
O*W*3—H31⋯F6^vi^	0.82 (4)	1.79 (4)	2.612 (4)	176
O*W*3—H32⋯F1^ii^	0.81 (7)	1.99 (7)	2.783 (4)	167

Symmetry codes: (i) 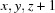; (ii) 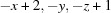; (iii) 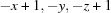; (iv) 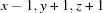; (v) 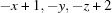; (vi) 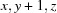; (vii) 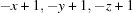; (viii) 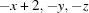.

## supplementary crystallographic information

### S1. Comment

Hybrid solids, containing both organic and inorganic entities, have diverse crystal structures which can influence their physicochemical properties. In type I solid hybrids, interactions between organic and inorganic networks are generally weak (*e.g.* hydrogen bonds or van der Waals' interactions), whilst in type II hybrids, covalent bonds are generally established between the metal of the inorganic moiety and the organic moiety. Type II hybrid materials usually exhibit better thermal stability than those of type I. Many chemical systems have been explored by conventional hydro- solvothermal synthesis or microwave heating. This work deals with a new aluminium fluoride salt of hybrid type I prepared under solvothermal conditions. Its structure contains isolated AlF_6_ distorted octahedra hydrogen bonded to propane-1,3-diamine dications and water molecules, together with fluoride ions which are also hydrogen bonded to the organic dications (Figures 2-4).

### S2. Experimental

The title compound was prepared from a starting mixture of Al(OH)_3_ (0.75 g) in 40% HF (0.8 ml) and ethanol (5 ml). 1,3- diaminopropane (1 ml) was added and mild hydrothermal conditions (463 K) were applied in a Teflon lined autoclave (25 mL). The resulting product was washed with ethanol and dried in air giving colorless single crystals of the title compound.

### S3. Refinement

All non-hydrogen atoms were refined with anisotropic displacement parameters. The H atoms of the water molecules were located using difference Fourier methods and their positional and isotropic displacement parameters refined. The H atoms of the organic dications were included in the refinement at calculated positions and refined with a common isotropic thermal parameter.

### Crystal data

**Table:**

(C_3_H_10_N_2_)_2_[AlF_6_]F·3H_2_O	*Z* = 2
*M**_r_* = 366.31	*F*(000) = 388
Triclinic, *P*1	*D*_x_ = 1.477 Mg m^−^^3^
Hall symbol: -P 1	Mo *K*α radiation, λ = 0.71073 Å
*a* = 9.825 (2) Å	Cell parameters from 32 reflections
*b* = 9.974 (3) Å	θ = 5.0–20.0°
*c* = 10.697 (2) Å	µ = 0.21 mm^−^^1^
α = 70.01 (2)°	*T* = 298 K
β = 67.89 (2)°	Platelet, colorless
γ = 59.77 (1)°	0.61 × 0.13 × 0.08 mm
*V* = 823.8 (3) Å^3^	

### Data collection

**Table:**

Siemens AED2 diffractometer	3046 reflections with *I* > 2σ(*I*)
Radiation source: fine-focus sealed tube	*R*_int_ = 0.000
Graphite monochromator	θ_max_ = 27.6°, θ_min_ = 2.1°
2θ/ω scan	*h* = −11→12
Absorption correction: gaussian (*SADABS*; Sheldrick, 1996)	*k* = −11→12
*T*_min_ = 0.968, *T*_max_ = 0.985	*l* = 0→13
3411 measured reflections	3 standard reflections every 120 min
3411 independent reflections	intensity decay: 4%

### Refinement

**Table:**

Refinement on *F*^2^	Primary atom site location: structure-invariant direct methods
Least-squares matrix: full	Secondary atom site location: difference Fourier map
*R*[*F*^2^ > 2σ(*F*^2^)] = 0.059	Hydrogen site location: inferred from neighbouring sites
*wR*(*F*^2^) = 0.183	H atoms treated by a mixture of independent and constrained refinement
*S* = 1.05	' *w* = 1/[σ^2^(*F*_o_^2^) + (0.1177*P*)^2^ + 0.6344*P*] where *P* = (*F*_o_^2^ + 2*F*_c_^2^)/3'
3411 reflections	(Δ/σ)_max_ = 0.009
215 parameters	Δρ_max_ = 0.62 e Å^−^^3^
0 restraints	Δρ_min_ = −0.54 e Å^−^^3^

### Special details

**Table:**

Geometry. All esds (except the esd in the dihedral angle between two l.s. planes) are estimated using the full covariance matrix. The cell esds are taken into account individually in the estimation of esds in distances, angles and torsion angles; correlations between esds in cell parameters are only used when they are defined by crystal symmetry. An approximate (isotropic) treatment of cell esds is used for estimating esds involving l.s. planes.
Refinement. Refinement of F^2^ against ALL reflections. The weighted *R*-factor *wR* and goodness of fit S are based on F^2^, conventional *R*-factors *R* are based on F, with F set to zero for negative F^2^. The threshold expression of F^2^ > 2sigma(F^2^) is used only for calculating *R*-factors(gt) *etc*. and is not relevant to the choice of reflections for refinement. *R*-factors based on F^2^ are statistically about twice as large as those based on F, and R– factors based on ALL data will be even larger.

### Fractional atomic coordinates and isotropic or equivalent isotropic displacement parameters (Å^2^)

**Table:**

	*x*	*y*	*z*	*U*_iso_*/*U*_eq_	
Al1	0.83033 (8)	−0.20234 (8)	0.29308 (6)	0.0238 (2)	
F1	1.0025 (2)	−0.3849 (2)	0.2527 (3)	0.0678 (6)	
F2	0.9474 (2)	−0.1577 (3)	0.35631 (17)	0.0496 (5)	
F3	0.9030 (2)	−0.1056 (3)	0.12502 (16)	0.0518 (5)	
F4	0.7092 (2)	−0.2378 (3)	0.2304 (2)	0.0575 (5)	
F5	0.6596 (2)	−0.0158 (2)	0.3295 (2)	0.0546 (5)	
F6	0.7625 (3)	−0.2962 (3)	0.4612 (2)	0.0747 (8)	
F7	0.80224 (19)	−0.13548 (18)	0.76019 (15)	0.0357 (4)	
N1	0.86118 (18)	0.02879 (18)	0.8720 (2)	0.0295 (4)	
H1A	0.8503	−0.0243	0.8272	0.054 (2)*	
H1B	0.8678	−0.0258	0.9567	0.054 (2)*	
H1C	0.9513	0.0429	0.8278	0.054 (2)*	
N2	0.26017 (18)	0.32258 (18)	0.9923 (2)	0.0333 (4)	
H2A	0.2747	0.2379	0.9690	0.054 (2)*	
H2B	0.1927	0.4094	0.9480	0.054 (2)*	
H2C	0.2178	0.3171	1.0825	0.054 (2)*	
N3	0.6255 (3)	0.0499 (3)	0.5761 (2)	0.0348 (5)	
H3A	0.6799	−0.0092	0.6407	0.054 (2)*	
H3B	0.6738	0.0038	0.5032	0.054 (2)*	
H3C	0.5238	0.0599	0.6089	0.054 (2)*	
N4	0.9607 (3)	0.3445 (2)	0.4048 (2)	0.0326 (5)	
H4A	0.9932	0.3030	0.4823	0.054 (2)*	
H4B	0.9584	0.4403	0.3719	0.054 (2)*	
H4C	1.0296	0.2828	0.3433	0.054 (2)*	
C1	0.7954 (3)	0.3567 (3)	0.4329 (3)	0.0355 (5)	
H1D	0.7567	0.4093	0.3501	0.054 (2)*	
H1E	0.7214	0.4199	0.5031	0.054 (2)*	
C2	0.7184 (3)	0.1845 (3)	0.8795 (3)	0.0308 (5)	
H2D	0.7159	0.2468	0.7874	0.054 (2)*	
H2E	0.7284	0.2406	0.9314	0.054 (2)*	
C3	0.6240 (3)	0.2082 (3)	0.5355 (3)	0.0334 (5)	
H3D	0.5688	0.2597	0.6144	0.054 (2)*	
H3E	0.5646	0.2727	0.4652	0.054 (2)*	
C4	0.5606 (3)	0.1665 (3)	0.9473 (3)	0.0312 (5)	
H4D	0.5485	0.1128	0.8945	0.054 (2)*	
H4E	0.5628	0.1034	1.0391	0.054 (2)*	
C5	0.7966 (3)	0.1947 (3)	0.4807 (3)	0.0312 (5)	
H5D	0.8544	0.1343	0.5524	0.054 (2)*	
H5E	0.8535	0.1387	0.4046	0.054 (2)*	
C6	0.4188 (3)	0.3281 (3)	0.9548 (3)	0.0351 (5)	
H6D	0.4199	0.3720	1.0223	0.054 (2)*	
H6E	0.4313	0.3976	0.8663	0.054 (2)*	
OW1	0.6507 (3)	0.2741 (3)	0.1872 (3)	0.0543 (6)	
H11	0.658 (7)	0.173 (3)	0.216 (5)	0.096 (17)*	
H12	0.536 (3)	0.334 (6)	0.214 (6)	0.12 (2)*	
OW2	1.0850 (3)	−0.3475 (3)	−0.0480 (3)	0.0480 (5)	
OW3	0.6830 (3)	0.5567 (4)	0.7114 (3)	0.0670 (8)	
H21	1.053 (6)	−0.325 (5)	0.020 (5)	0.063 (14)*	
H22	1.160 (6)	−0.322 (6)	−0.080 (5)	0.073 (14)*	
H31	0.712 (5)	0.602 (5)	0.633 (4)	0.052 (10)*	
H32	0.767 (7)	0.507 (6)	0.735 (6)	0.090 (17)*	

### Atomic displacement parameters (Å^2^)

**Table:**

	*U*^11^	*U*^22^	*U*^33^	*U*^12^	*U*^13^	*U*^23^
Al1	0.0238 (3)	0.0282 (4)	0.0245 (3)	−0.0153 (3)	−0.0087 (2)	−0.0013 (2)
F1	0.0399 (10)	0.0365 (9)	0.1124 (18)	−0.0088 (8)	−0.0130 (11)	−0.0167 (10)
F2	0.0553 (10)	0.0851 (13)	0.0371 (8)	−0.0519 (10)	−0.0159 (7)	−0.0044 (8)
F3	0.0537 (10)	0.0853 (14)	0.0284 (8)	−0.0501 (10)	−0.0160 (7)	0.0141 (8)
F4	0.0516 (11)	0.0991 (16)	0.0566 (11)	−0.0513 (11)	−0.0063 (8)	−0.0322 (10)
F5	0.0335 (9)	0.0417 (9)	0.0857 (14)	−0.0117 (7)	−0.0063 (9)	−0.0258 (9)
F6	0.0929 (17)	0.117 (2)	0.0379 (9)	−0.0847 (17)	−0.0185 (10)	0.0246 (11)
F7	0.0399 (8)	0.0398 (8)	0.0357 (8)	−0.0209 (7)	−0.0141 (6)	−0.0054 (6)
N1	0.0270 (9)	0.0363 (10)	0.0326 (10)	−0.0193 (8)	−0.0110 (8)	−0.0020 (8)
N2	0.0315 (10)	0.0368 (11)	0.0344 (10)	−0.0175 (9)	−0.0079 (8)	−0.0058 (8)
N3	0.0325 (10)	0.0493 (12)	0.0352 (10)	−0.0257 (10)	−0.0110 (8)	−0.0065 (9)
N4	0.0341 (11)	0.0329 (10)	0.0366 (10)	−0.0194 (9)	−0.0113 (8)	−0.0027 (8)
C1	0.0297 (11)	0.0318 (12)	0.0465 (14)	−0.0128 (10)	−0.0151 (10)	−0.0035 (10)
C2	0.0329 (12)	0.0322 (12)	0.0343 (11)	−0.0206 (10)	−0.0095 (9)	−0.0027 (9)
C3	0.0271 (11)	0.0391 (13)	0.0379 (12)	−0.0158 (10)	−0.0099 (9)	−0.0071 (10)
C4	0.0307 (11)	0.0317 (11)	0.0367 (12)	−0.0184 (10)	−0.0101 (9)	−0.0031 (9)
C5	0.0249 (10)	0.0349 (12)	0.0374 (12)	−0.0144 (9)	−0.0082 (9)	−0.0082 (9)
C6	0.0303 (12)	0.0337 (12)	0.0475 (14)	−0.0162 (10)	−0.0118 (10)	−0.0086 (10)
OW1	0.0534 (13)	0.0473 (12)	0.0579 (13)	−0.0279 (11)	−0.0084 (11)	−0.0017 (10)
OW2	0.0400 (11)	0.0468 (12)	0.0585 (14)	−0.0144 (10)	−0.0093 (10)	−0.0217 (10)
OW3	0.0425 (13)	0.0716 (17)	0.0512 (14)	−0.0207 (12)	−0.0061 (11)	0.0162 (12)

### Geometric parameters (Å, º)

**Table:**

Al1—F6	1.7690 (19)	N3—H3B	0.8900
Al1—F4	1.7832 (17)	N3—H3C	0.8900
Al1—F3	1.7929 (16)	N4—C1	1.483 (3)
Al1—F1	1.803 (2)	N4—H4A	0.8900
Al1—F2	1.8126 (16)	N4—H4B	0.8900
Al1—F5	1.8130 (19)	N4—H4C	0.8900
F1—F3	2.499 (3)	C1—C5	1.514 (3)
F1—F4	2.556 (3)	C1—H1D	0.9700
F1—F6	2.570 (4)	C1—H1E	0.9700
F1—F2	2.586 (3)	C2—C4	1.518 (3)
F2—F3	2.507 (2)	C2—H2D	0.9700
F2—F5	2.527 (3)	C2—H2E	0.9700
F2—F6	2.529 (3)	C3—C5	1.521 (3)
F3—F4	2.556 (2)	C3—H3D	0.9700
F3—F5	2.559 (3)	C3—H3E	0.9700
F4—F5	2.529 (3)	C4—C6	1.513 (3)
F4—F6	2.529 (3)	C4—H4D	0.9700
F5—F6	2.522 (3)	C4—H4E	0.9700
F5—H11	1.85 (3)	C5—H5D	0.9700
N1—C2	1.481 (3)	C5—H5E	0.9700
N1—H1A	0.8900	C6—H6D	0.9700
N1—H1B	0.8900	C6—H6E	0.9700
N1—H1C	0.8900	OW1—H11	0.925 (19)
N2—C6	1.481 (3)	OW1—H12	0.95 (2)
N2—H2A	0.8900	OW2—H21	0.74 (5)
N2—H2B	0.8900	OW2—H22	0.82 (5)
N2—H2C	0.8900	OW3—H31	0.83 (4)
N3—C3	1.482 (3)	OW3—H32	0.81 (6)
N3—H3A	0.8900		
			
F6—Al1—F4	90.79 (10)	H1A—N1—H1C	109.5
F6—Al1—F3	177.96 (9)	H1B—N1—H1C	109.5
F4—Al1—F3	91.25 (9)	C6—N2—H2A	109.5
F6—Al1—F1	92.04 (13)	C6—N2—H2B	109.5
F4—Al1—F1	90.93 (11)	H2A—N2—H2B	109.5
F3—Al1—F1	88.04 (11)	C6—N2—H2C	109.5
F6—Al1—F2	89.84 (9)	H2A—N2—H2C	109.5
F4—Al1—F2	177.63 (11)	H2B—N2—H2C	109.5
F3—Al1—F2	88.11 (8)	C3—N3—H3A	109.5
F1—Al1—F2	91.33 (11)	C3—N3—H3B	109.5
F6—Al1—F5	89.49 (12)	H3A—N3—H3B	109.5
F4—Al1—F5	89.36 (10)	C3—N3—H3C	109.5
F3—Al1—F5	90.41 (11)	H3A—N3—H3C	109.5
F1—Al1—F5	178.44 (10)	H3B—N3—H3C	109.5
F2—Al1—F5	88.36 (10)	C1—N4—H4A	109.5
F3—F1—F4	60.73 (8)	C1—N4—H4B	109.5
F3—F1—F6	89.25 (9)	H4A—N4—H4B	109.5
F4—F1—F6	59.11 (8)	C1—N4—H4C	109.5
F3—F1—F2	59.05 (7)	H4A—N4—H4C	109.5
F4—F1—F2	88.70 (8)	H4B—N4—H4C	109.5
F6—F1—F2	58.74 (8)	N4—C1—C5	111.0 (2)
F3—F2—F5	61.11 (8)	N4—C1—H1D	109.4
F3—F2—F6	90.00 (8)	C5—C1—H1D	109.4
F5—F2—F6	59.83 (8)	N4—C1—H1E	109.4
F3—F2—F1	58.74 (8)	C5—C1—H1E	109.4
F5—F2—F1	90.00 (8)	H1D—C1—H1E	108.0
F6—F2—F1	60.32 (9)	N1—C2—C4	111.36 (18)
F1—F3—F2	62.21 (9)	N1—C2—H2D	109.4
F1—F3—F4	60.74 (8)	C4—C2—H2D	109.4
F2—F3—F4	90.47 (7)	N1—C2—H2E	109.4
F1—F3—F5	91.25 (8)	C4—C2—H2E	109.4
F2—F3—F5	59.82 (8)	H2D—C2—H2E	108.0
F4—F3—F5	59.25 (7)	N3—C3—C5	110.7 (2)
F5—F4—F6	59.81 (9)	N3—C3—H3D	109.5
F5—F4—F3	60.43 (8)	C5—C3—H3D	109.5
F6—F4—F3	88.90 (7)	N3—C3—H3E	109.5
F5—F4—F1	90.64 (8)	C5—C3—H3E	109.5
F6—F4—F1	60.72 (10)	H3D—C3—H3E	108.1
F3—F4—F1	58.52 (7)	C6—C4—C2	109.45 (19)
F6—F5—F4	60.10 (9)	C6—C4—H4D	109.8
F6—F5—F2	60.13 (8)	C2—C4—H4D	109.8
F4—F5—F2	90.65 (8)	C6—C4—H4E	109.8
F6—F5—F3	89.00 (9)	C2—C4—H4E	109.8
F4—F5—F3	60.32 (7)	H4D—C4—H4E	108.2
F2—F5—F3	59.07 (7)	C1—C5—C3	110.8 (2)
Al1—F5—H11	120.3 (17)	C1—C5—H5D	109.5
F6—F5—H11	160.8 (17)	C3—C5—H5D	109.5
F4—F5—H11	120.6 (16)	C1—C5—H5E	109.5
F2—F5—H11	101.0 (17)	C3—C5—H5E	109.5
F3—F5—H11	76.8 (17)	H5D—C5—H5E	108.1
F5—F6—F2	60.03 (8)	N2—C6—C4	112.33 (19)
F5—F6—F4	60.09 (9)	N2—C6—H6D	109.1
F2—F6—F4	90.59 (8)	C4—C6—H6D	109.1
F5—F6—F1	90.47 (8)	N2—C6—H6E	109.1
F2—F6—F1	60.94 (8)	C4—C6—H6E	109.1
F4—F6—F1	60.16 (9)	H6D—C6—H6E	107.9
C2—N1—H1A	109.5	H11—OW1—H12	100 (5)
C2—N1—H1B	109.5	H21—OW2—H22	100 (5)
H1A—N1—H1B	109.5	H31—OW3—H32	102 (5)
C2—N1—H1C	109.5		

### Hydrogen-bond geometry (Å, º)

**Table:**

*D*—H···*A*	*D*—H	H···*A*	*D*···*A*	*D*—H···*A*
N1—H1*A*···F7	0.89	1.81	2.696 (3)	171
N1—H1*B*···F3^i^	0.89	1.79	2.663 (3)	166
N1—H1*C*···F2^ii^	0.89	2.08	2.826 (3)	141
N1—H1*C*···F3^ii^	0.89	2.09	2.796 (3)	136
N2—H2*A*···F4^iii^	0.89	2.08	2.657 (3)	122
N2—H2*B*···O*W*2^iv^	0.89	2.11	2.804 (3)	134
N2—H2*C*···F7^v^	0.89	2.04	2.724 (3)	132
N3—H3*A*···F7	0.89	1.79	2.677 (3)	176
N3—H3*B*···F5	0.89	2.00	2.792 (3)	148
N3—H3*C*···F5^iii^	0.89	1.89	2.753 (4)	162
N4—H4*A*···F2^ii^	0.89	1.90	2.757 (3)	161
N4—H4*B*···F1^vi^	0.89	1.92	2.776 (4)	162
N4—H4*C*···F7^ii^	0.89	1.84	2.724 (3)	169
O*W*1—H11···F5	0.92	1.85	2.743 (3)	161
O*W*1—H12···O*W*3^vii^	0.95	1.85	2.789 (5)	173
O*W*2—H21···F1	0.74	2.27 (5)	2.943 (3)	152 (5)
O*W*2—H22···O*W*1^viii^	0.82 (7)	1.98	2.785 (5)	170
O*W*3—H31···F6^vi^	0.82 (4)	1.79 (4)	2.612 (4)	176
O*W*3—H32···F1^ii^	0.81 (7)	1.99 (7)	2.783 (4)	167

Symmetry codes: (i) *x*, *y*, *z*+1; (ii) −*x*+2, −*y*, −*z*+1; (iii) −*x*+1, −*y*, −*z*+1; (iv) *x*−1, *y*+1, *z*+1; (v) −*x*+1, −*y*, −*z*+2; (vi) *x*, *y*+1, *z*; (vii) −*x*+1, −*y*+1, −*z*+1; (viii) −*x*+2, −*y*, −*z*.
