# Supplementary figures and images for: Crystal structure of bis­(propane-1,3-di­ammonium) hexa­fluorido­aluminate fluoride trihydrate
Source: Acta Crystallogr Sect E Struct Rep Online. 2014 Aug 30;70(Pt 9):m335–6. doi: 10.1107/S1600536814018844 (PMC4186155; doi:10.1107/S1600536814018844)

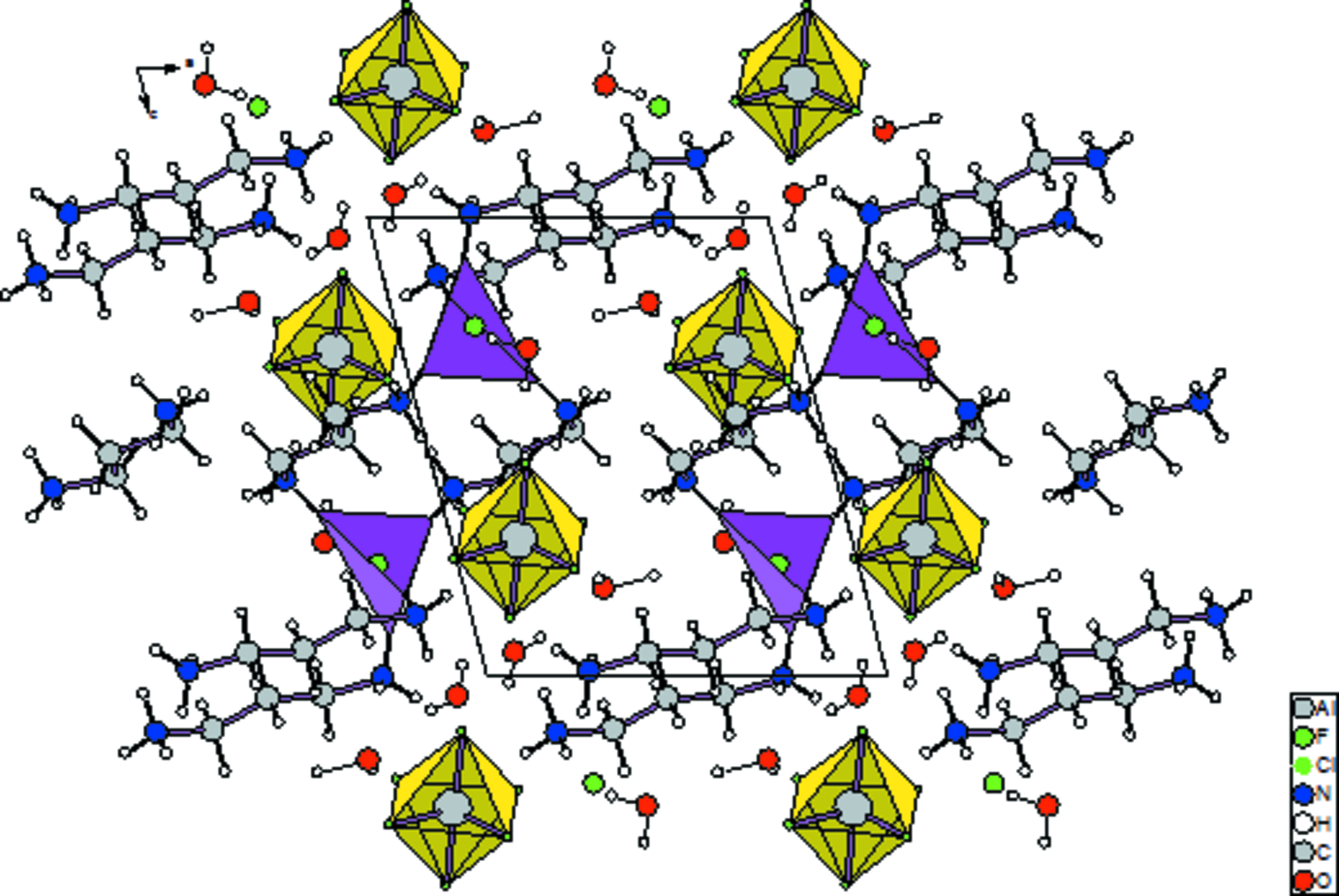

Supplement: Supplementary file 3 [file e-70-0m335-fig1.tif]

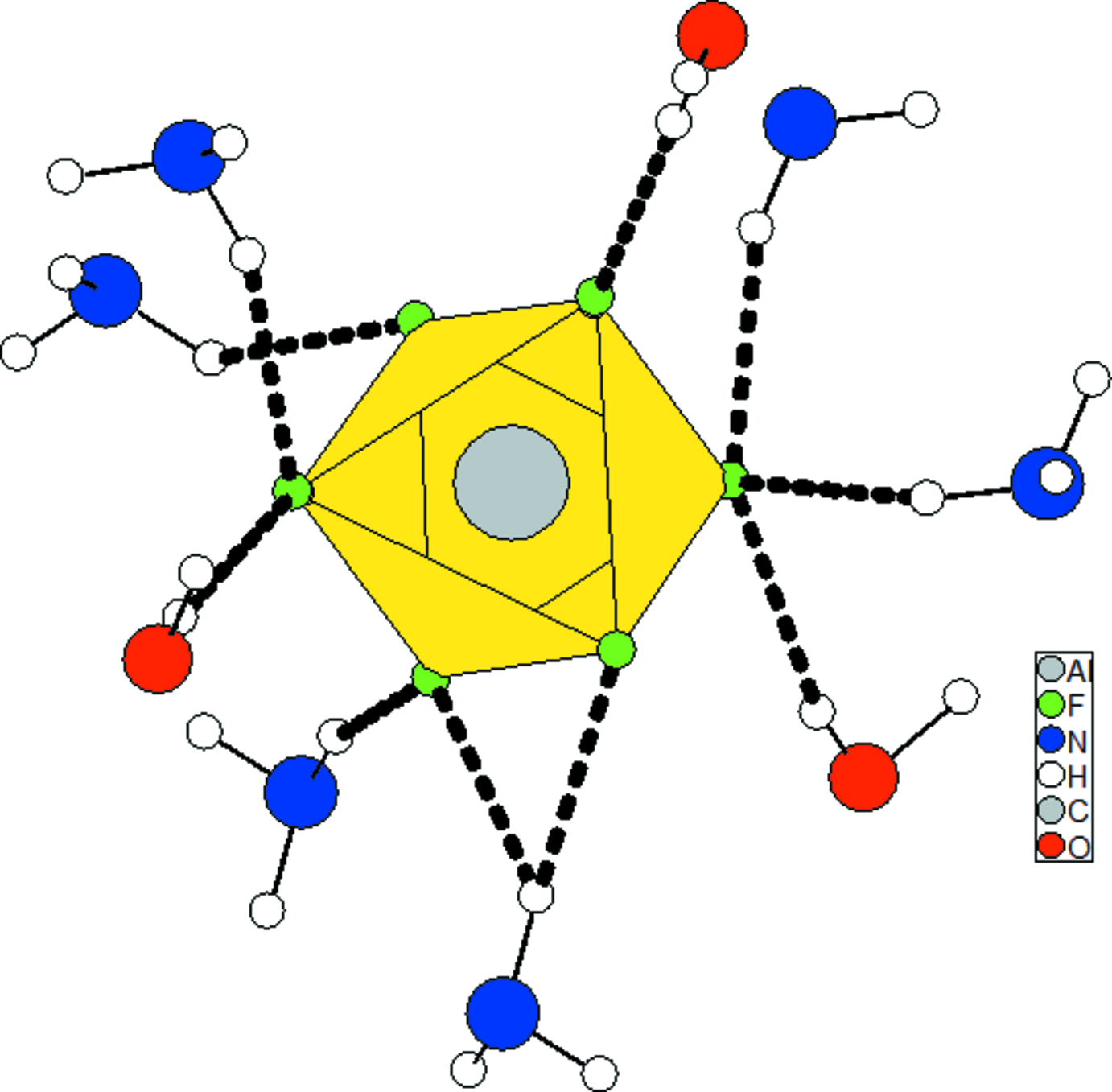

Supplement: Supplementary file 4 [file e-70-0m335-fig2.tif]

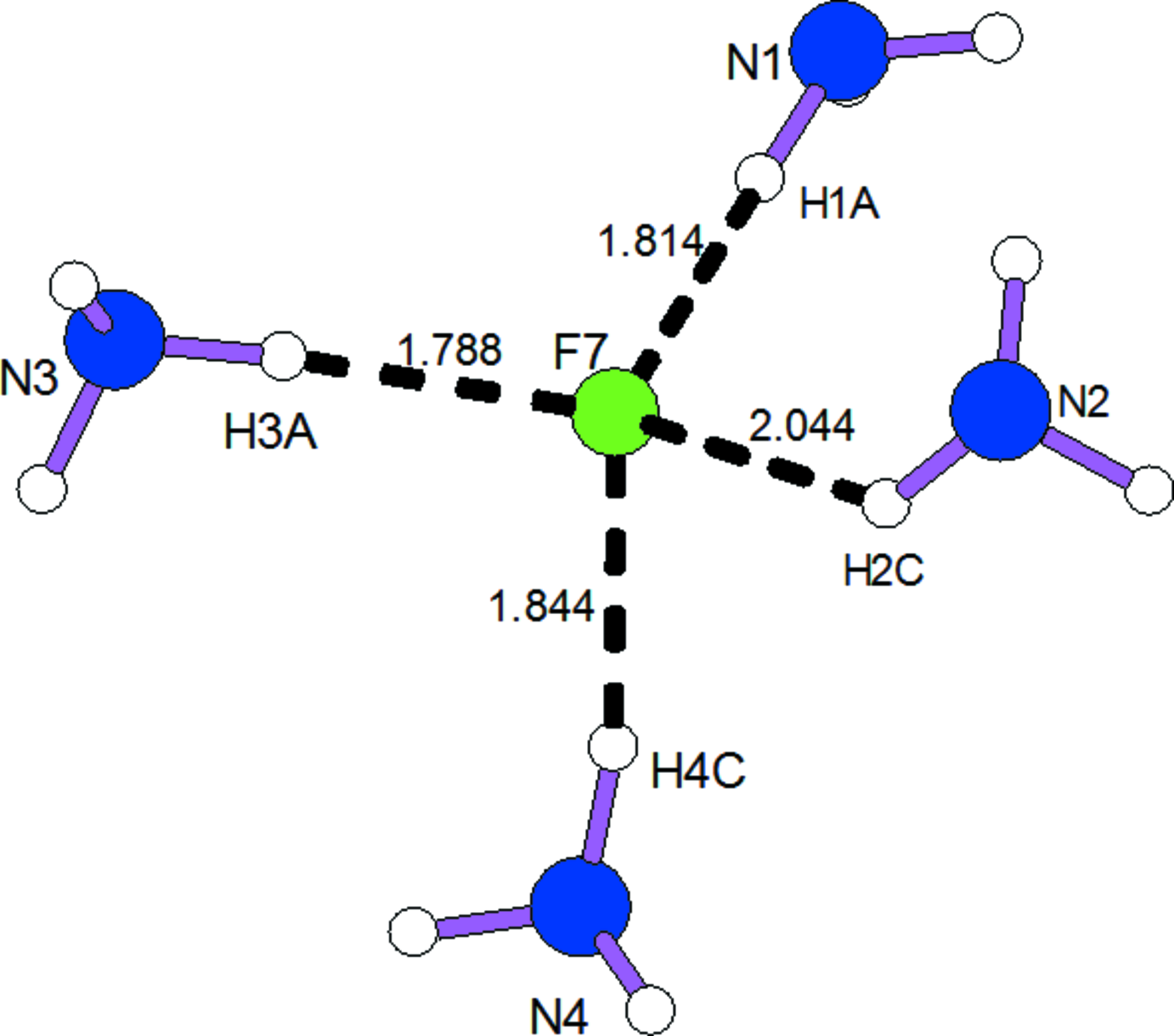

Supplement: Supplementary file 5 [file e-70-0m335-fig3.tif]

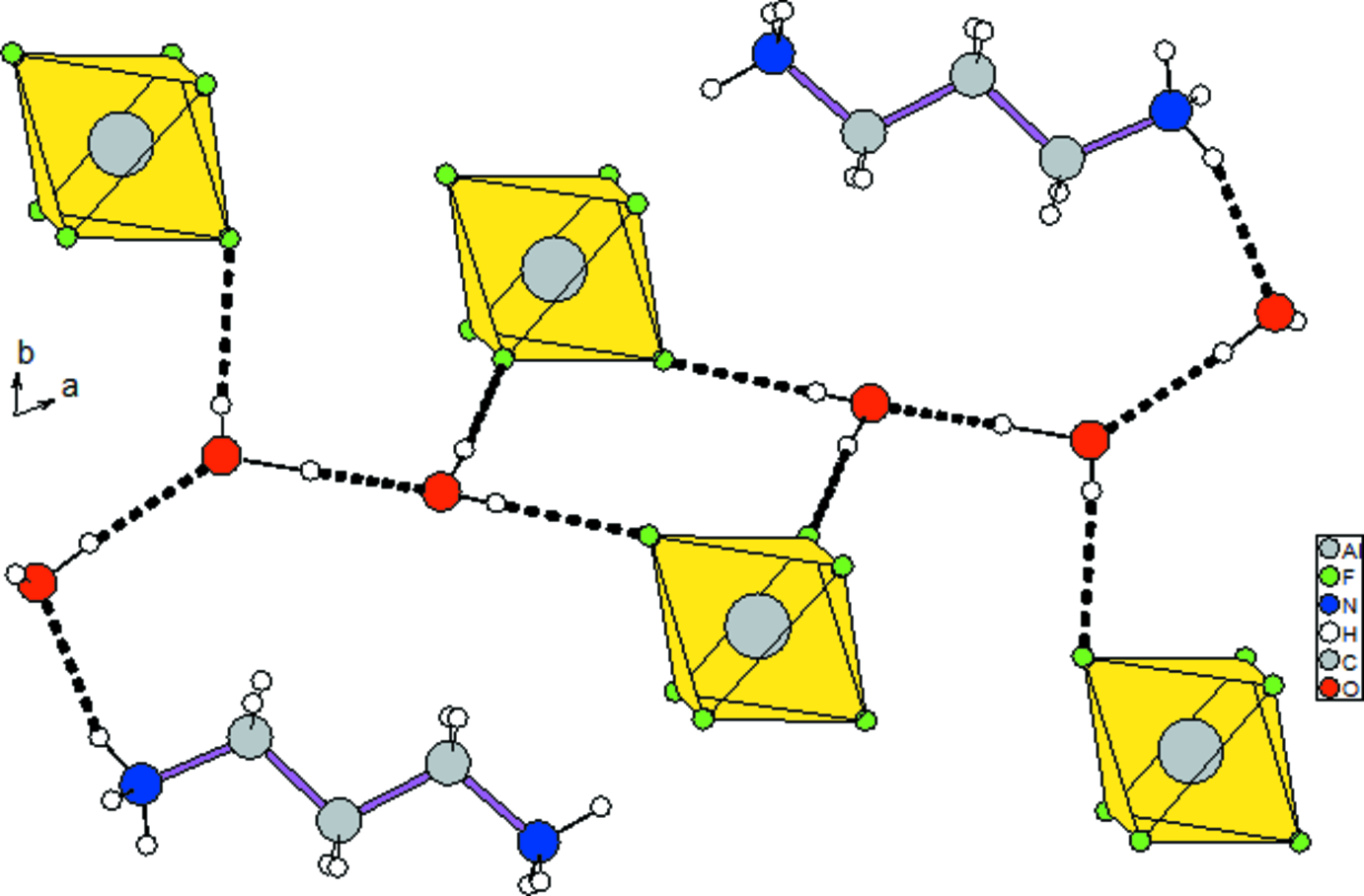

Supplement: Supplementary file 6 [file e-70-0m335-fig4.tif]
